# A compact tunable quadrupole lens for brighter and sharper ultra-fast electron diffraction imaging

**DOI:** 10.1038/s41598-019-39208-z

**Published:** 2019-03-26

**Authors:** Xi Yang, Lihua Yu, Victor Smaluk, Guimei Wang, Yoshitreu Hidaka, Timur Shaftan, Lewis Doom, Danny Padrazo, Junjie Li, Mikhail Fedurin, Weishi Wan, Yimei Zhu

**Affiliations:** 1 Brookhaven National Laboratory, Upton, NY 11973 USA; 2 ShanghaiTech University, Shanghai, China

## Abstract

In this article, we report our proof-of-principle design and experimental commissioning of a broadly tunable and low-cost transverse focusing lens system for MeV-energy electron beams. The lens system based on electromagnetic (EM) quadrupoles has been built as a part of the existing instrument for ultra-fast electron diffraction (UED) experiments at the Accelerator Test Facility II (ATF-II) at Brookhaven National Laboratory (BNL). We experimentally demonstrated the independent control of the size and divergence of the beam with the charge ranging from 1 to 13 pC. The charge density and divergence of the beam at the sample are the most important factors determining the quality of the Bragg-diffraction image (BDI). By applying the Robust Conjugate Directional Search (RCDS) algorithm for online optimization of the quadrupoles, the transverse beam size can be kept constant down to 75 µm from 1 to 13 pC. The charge density is nearly two orders of magnitude higher than the previously achieved value using a conventional solenoid. Using the BDI method we were able to extract the divergence of the beam in real-time and apply it to the emittance measurement for the first time. Our results agree well with simulations and with the traditional quadrupole scan method. The real-time divergence measurement opens the possibility of online optimization of the beam divergence (<0.2 mrad) at the sample with the increased beam charge. This optimization is crucial for the future development of single-shot ultra-fast electron microscope (UEM). Finally, we demonstrated BDI with significant improvement, up to 3 times higher peak intensity and 2 times sharper Bragg-diffraction peaks at 13 pC. The charge is now limited by the laser power and increasing charge may improve the quality of BDI further. The capability we demonstrated here provides us with opportunities for new sciences using near-parallel, bright and ultrafast electron beams for single-shot imaging, to directly visualize the dynamics of defects and nanostructured materials, or even record molecular movie, which are impossible using present electron-beam technologies.

## Introduction

An ultra-fast electron diffraction facility delivering up to 0.8·10^8^ electrons (13 pC) in a single-shot mode with the electron energy of 3.3MeV and the temporal resolution of 100 fs to 1 ps^1–8^ represents a unique opportunity of simultaneous high temporal and spatial resolution for studies of many processes in physics, chemistry and biology. Examples include resolving the structure of proteins that cannot be crystallized or non-periodic structures^9^. By employing an accelerator-based radio-frequency (RF) photoinjector as the MeV electron source for the time-resolved electron diffraction, UED takes advantage of the strong interaction between electrons and matter and minimizes space charge problems. Due to the almost 1000-fold shorter wavelength of electrons compared to X-rays, UED can resolve much finer structural details enabling us to see how atoms in molecules move and make molecular movies of ultrafast chemical reactions. Therefore, putting both XFEL and UED together will provide a more complete picture in groundbreaking studies of all kinds of complex dynamic processes in nature^10^.

There are many technical challenges which must be overcome before mega-electron-volt UED can be turned into a significant tool in ultrafast science and technology. Most importantly, a much brighter electron source is required than what is currently available. Quadrupoles are known to have very strong focusing capability, especially for high-energy electron beams due to their focusing strength being inversely proportional to the momentum^11^. In comparison, the focusing power of a round magnetic lens is inversely proportional to the momentum squared^12^. Therefore, the focusing system based on quadrupoles can be made much more compact and lower cost. To overcome the property of a quadrupole focusing in one direction while defocusing in the other, at least two quadrupoles with opposite polarities are necessary to form a lens that focuses the beam in both transverse directions. A multiplet of quadrupoles, including quadruplet and quintuplet, can form a lens with more desirable properties, such as rotational symmetry of the paraxial rays and widely tunable focal length. Two sets of the quadrupole multiplets are needed to place the waist of the beam at the sample and tune the spot size simultaneously. In this article, we report our proof-of-principle design and experimental commissioning of broadly tunable and low-cost transverse focusing lens system for MeV-energy electron beams. Such a system based on quadrupole multiplets has been built as a part of the existing instrument for UED experiments at ATF-II, BNL. It has been successfully commissioned with the capability of generating 3.3 MeV electron bunches with 13 pC charge with the focused beam size 75 µm^1–3^.

The qualities (signal-to-noise ratio and resolution) of diffraction image are primarily determined by the charge density and divergence of the electron beam at the sample. The quadrupole system provides independent control over the size and divergence of the beam at the sample. We demonstrated that when focused to 75 µm the charge density of the beam is nearly two orders of magnitude higher than achieved previously using conventional techniques of beam focusing by a solenoid only. Furthermore, transverse beam sizes can be kept constant at about 75 µm from 1 to 13 pC *via* RCDS online optimization^13^.

We focused on improving the intensity and resolution of the diffraction peaks *via* optimizing the quadrupoles in two different configurations. In the first configuration, the sample was placed upstream of the quadrupoles; therefore, the solenoid was used to focus the electron beam on the sample, and the quadrupoles were applied to focus the diffraction image on the detector. This experiment is leading to the future ultrafast electron imaging upgrade. We demonstrated a factor of 2 to 3 increase of the BDI intensity. In this paper, we will concentrate on the second configuration where the sample is placed downstream of the quadrupoles in the diagnostic chamber. The role of the quadrupoles is to minimize the divergence of the beam as well as reduce its size on the sample. The source divergence is the main factor (together with other parameters such as energy spread of the beam) determining the diffraction peak width. This experiment succeeded for the first time measuring the divergence and emittance of the beam in real time *via* the BDI method. The BDI method has advantages compared with the conventional method of emittance measurement, which requires a quadrupole scan. We present the preliminary result of the measured emittance, which agrees well with simulations and with the conventional quadrupole scan^14^. The real-time measurement of the beam divergence makes online optimization of the beam opening angle possible with different charges.

In both configurations, we have achieved significant improvements in the brightness and sharpness of Bragg-diffraction peaks. Furthermore, it was demonstrated that a single quadrupole fed by a bi-polar power supply can compensate environmental perturbations, such as the remnant magnetic field, x-y asymmetry of the laser spot on cathode, and the misaligned beam trajectory, etc., to achieve the best controllable azimuthal symmetry of diffraction images.

## Results

### Beam focusing and diagnostics

**Figure 1:**
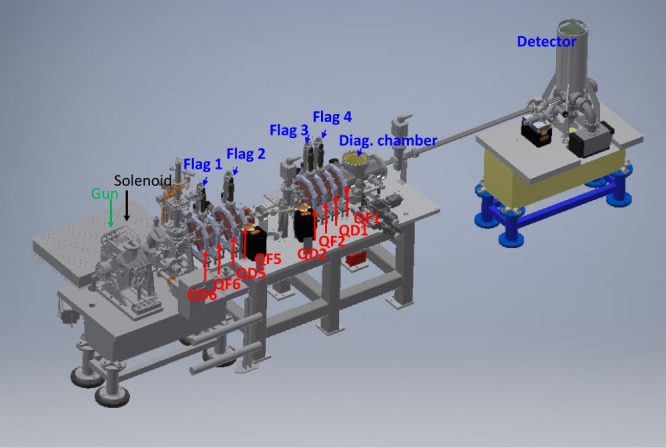
UED beamline with positions of the YAG screens and detector.

The layout of the UED beamline is shown in Fig. 1. After an electron bunch generated by a 100 fs UV laser pulse exits from the photocathode RF gun, it is focused by a tunable quadrupole lens system down to a small beam size or a small beam divergence, to probe the sample. The quadrupole lenses focusing the beam horizontally are marked as QF, and defocusing the beam horizontally are marked as QD. The system is flexible enough to switch between minimum beam size (≤75 µm) and minimum beam divergence (<0.2mrad) at the sample depending on the type of the experiments.

The key part of the design is the computer simulation of the beam dynamics for optimizing the tunable quadrupole system delivering the best-focused electron beam up to tens of pico-coulombs to the sample. The quadrupole system is able to compensate the space charge defocusing effects.

Based on the simulation using Impact-T code^15^, which takes the space-charge effects into proper considerations, the bunch length increases from 150 fs to 1.1 ps and the energy spread increases from 1.5·10^−3^ to 1.3·10^−2^ with the increase of the beam charge from 1 to 13 pC, transverse beam sizes or divergences still can be kept constant due to the broad-range tunability of the quadrupoles^1–3^. The 13 pC is limited by the available power of the drive laser.

Four removable flags with YAG screens were used to diagnose the electron beam size and trajectory along the beamline. The spatial resolution is 50 μm/pixel. An additional YAG screen is installed inside the diagnostic chamber with the resolution of 6.9 μm/pixel (Fig. 1). The screen in the diagnostic chamber was used to minimize the beam size at the sample.

**Figure 2:**
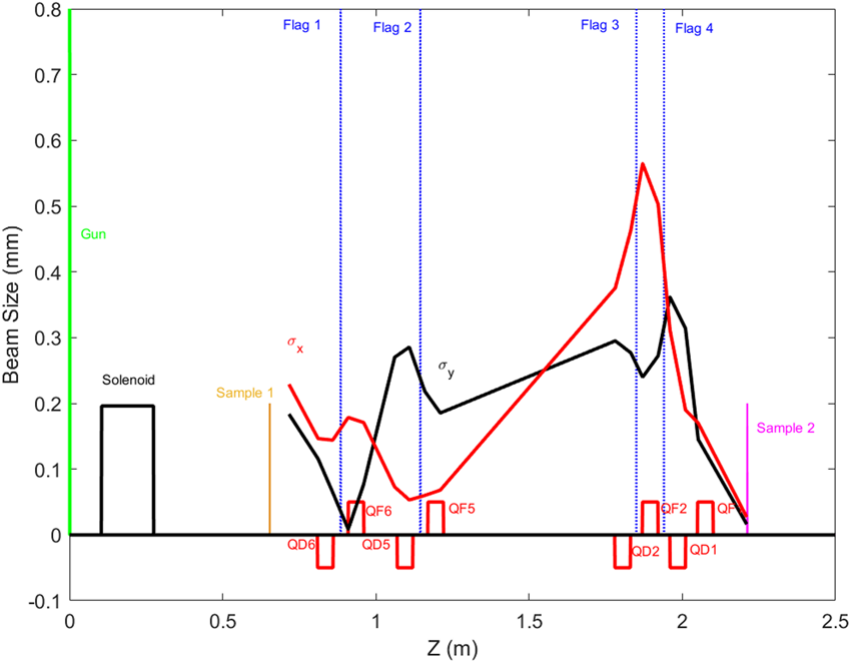
Horizontal σ_x_ and vertical σ_y_ beam sizes along the beamline. Locations of the beamline elements are also shown.

Figure 2 shows the horizontal and vertical beam sizes plotted as red and black curves, respectively, along the beamline with 10 pC beam charge. The longitudinal positions of quadrupoles (red squares), diagnostic beam profile monitors (vertical blue lines), sample in configuration 1 (vertical orange line), and sample in configuration 2 (vertical magenta line) are plotted at the bottom of the graph. Quadrupole systems can make a rotationally symmetric beam at the sample despite different initial beam conditions. Since the beam size at the diagnostic chamber is much smaller than beam sizes at other flags, we use an objective camera lens with higher magnification for the YAG screen located in the diagnostic chamber to increase the resolution to 6.9 μm/pixel.

### Minimizing beam size *via* RCDS online optimization

The quality of BDI is determined by both the charge density and divergence of the electron beam at the sample position. In cases when the sample size *σ*_sample_ is smaller than the beam size *σ*_beam_, it will be determined mainly by the charge density (beam sizes). Vice versa, if *σ*_sample_ is larger than *σ*_beam,_ it will be determined by the divergence of the beam. Therefore, we should choose either the size or the divergence of the beam as the target function of online optimization for the best BDI quality, respectively. The beam divergence can be precisely measured *via* the BDI method, which will be described later in the paper. In the first test of the lens system, we choose the combination $\sqrt{{\sigma }_{x}^{2}+{\sigma }_{y}^{2}}$ of beam sizes at the diagnostic chamber as the target function to be minimized *via* the RCDS online optimization. We demonstrated that the beam size can be kept nearly constant at different charges, even when the emittance increases with the charge. We compared horizontal *σ*_*x*_ and vertical *σ*_*y*_ beam sizes in the following two cases:

1. The quadrupole strengths are maintained at values optimized for the high beam charge (blue curves in Fig. 3).

**Figure 3:**
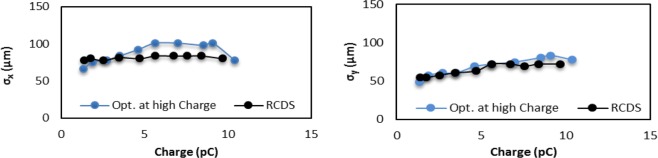
Horizontal beam size *σ*_*x*_ (left) and vertical beam size *σ*_*y*_ (right) at *Ф*_RF_ = 30°.
2. The quadrupole strengths are optimized at each charge (black curves in Fig. 3).

*σ*_*x*_ is shown in the left plot and *σ*_*y*_ is shown in the right plot. The beam aspect ratio deviates from 1 at lower charge because the target function does not take it into account. With the proper choice of the target function we can optimize the quadrupoles to maintain a constant beam size with the increased beam charge in spite of increased divergence and emittance. The resulting charge density is two orders of magnitude higher than the previously achieved value using a conventional solenoid.

### BDI Optimization in projector-lens-less configuration

In this case, the sample is placed in the diagnostic chamber downstream of the quadrupoles. There is no lens between the sample and the detector, so we call it projector-lens-less configuration. Therefore, there is no magnification. The sample is a polycrystalline Au film deposited on a carbon transmission electron microscope (TEM) grid several nanometers thick. The sample is 3 mm in diameter, 30 nm thick, and has a grain size in the range of tens of nanometers. The grid has a negligible contribution to the electron diffraction.

The BDI peaks are formed through the summation of the intensity distribution of all diffracted electrons. The diffraction pattern of a single electron is determined by the constructive interference governed by Bragg’s law 2*d* sin *θ* = *nλ*, where *θ* is the incident angle, *d* is the crystal interplanar distance, *λ* is the deBroglie wavelength, *n* is a positive integer. In our experimental condition *σ*_sample_ > *σ*_beam_, the whole beam is passing through the sample and the BDI is less sensitive to the beam size making the beam divergence critical. The BDI peak width is determined by the peak broadening due to the sample properties, the energy spread and the angular divergence of the electron beam. The narrower the peak width, the better is the image resolution. The energy spread $\frac{{\rm{\Delta }}{{\rm E}}}{{{\rm E}}}$ of the electron beam is in the order of 10^−3^ to 10^−2^ with the charge range from 1 to 13 pC. We estimate that the energy spread of the electron beam contributes only several (up to tens) microradians to the BDI peak width, much less than the diffraction peak width. The grain size and local strain of the sample also contribute to the peak broadening. In our case, the estimated peak broadening due to the sample properties is tens of µrad or less^16^. The BDI on the detector is the summation of the intensity distribution of all the electrons (~10^8^) in the beam. When the divergence of the beam is larger than the peak broadening due to the sample properties, the beam divergence becomes the main factor determining the diffraction peak width. Therefore, the diffraction peak width can be used to measure the divergence of the beam. We call this the BDI method.

**Figure 4:**
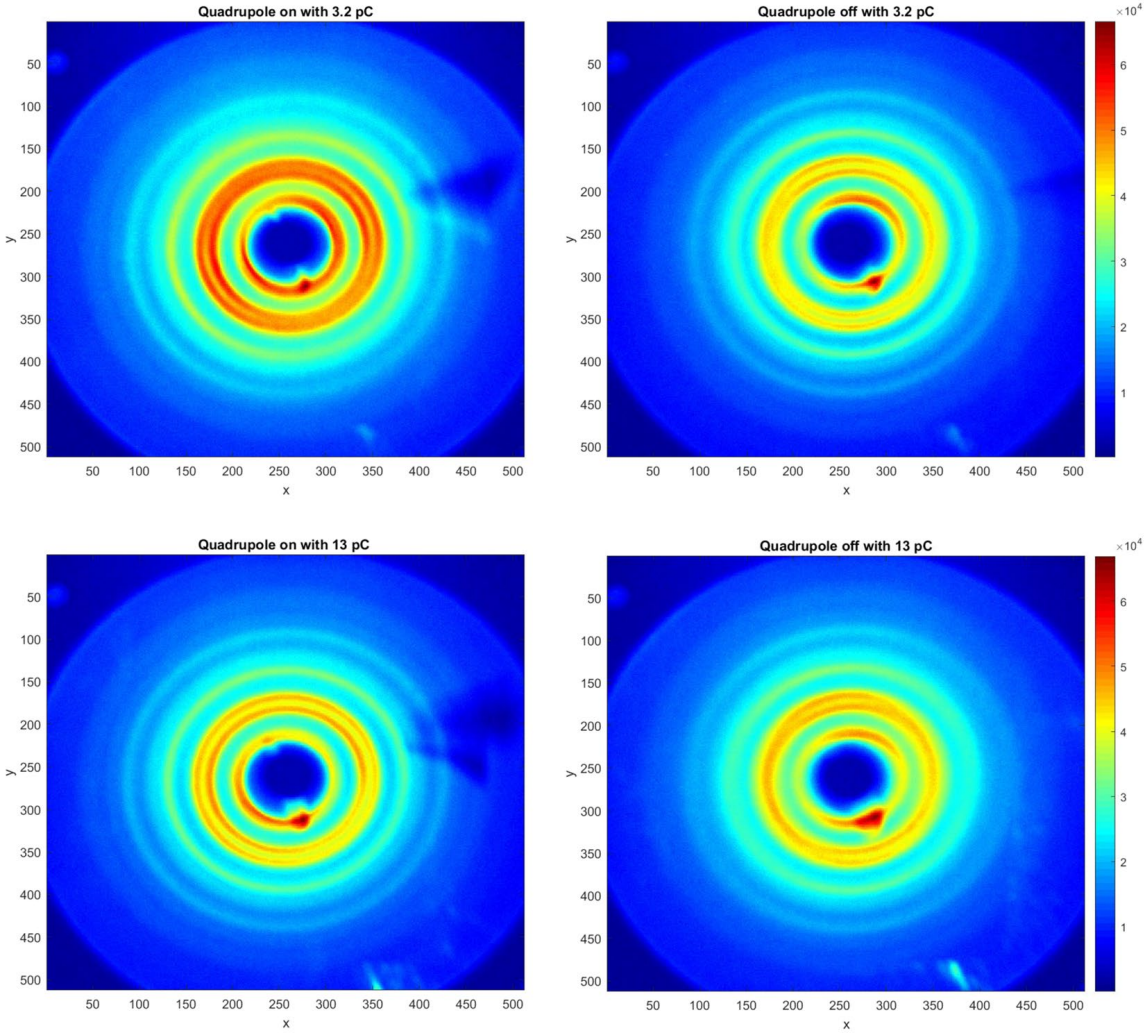
BDI at 3.2 pC (top row) and 13.0 pC (bottom row) with (left column) and without (right column) quadrupoles. We normalize the maximum intensities of all images to the same level.

From the above analysis, it is important for us to focus on improving the resolution of BDI via minimizing the beam divergence by optimizing the quadrupoles. The beam divergence was kept constant when the charge increases from 1 to 13 pC, limited by the drive laser energy. Only the beam size varied with the emittance. The enhancement of the BDI quality is shown in Fig. 4. When comparing the left column (with quadrupoles) to the right column (without quadrupoles) at the beam charge of 3.2 pC (top row) and 13.0 pC (bottom row). The BDI quality enhancement becomes more significant for the high-charge beam. Minimizing the divergence of a high-charge beam is important for the future development of single-shot UED^17^, where a well-compensated space-charge effect is required.

**Figure 5:**
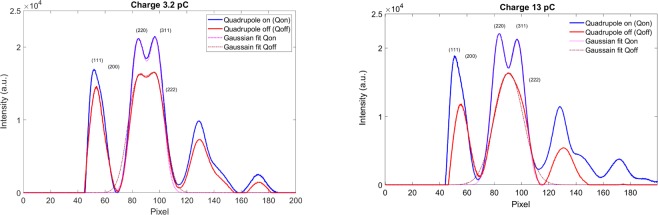
Comparison of the BD intensity distribution *via* a 360° averaging after the standard background subtraction and ring distortion correction to the image. Blue curves are with and red curves are without optimized quadrupoles.

We performed detailed analysis of the BD intensity distribution *via* a 360° averaging. The results are presented in Fig. 5. There are two different cases: with quadrupoles (blue curves) and without quadrupoles (red curves) at the beam charge of 3.2 pC (left) and 13.0 pC (right). We analyzed the profile along the horizontal axis between pixels 68 and 112, which covers the two highest-intensity diffraction peaks with Miller indexes (220) and (311). For the case with optimized quadrupoles, double-Gaussian distribution (magenta curves) fits the experimental data very well. Peak widths for both Miller indexes (220) and (311) are equal to 166 µrad at 3.2 pC. At 13.0 pC, both peak widths are 172 µrad.

Without quadrupoles, at 3.2 pC it is still possible to separate the two peaks (brown curves). The fitted peak widths are equal to 212 µrad. However, at 13.2 pC, it is no longer possible to separate the two peaks, so we fitted to one Gaussian peak and the width is 336 µrad.

The sharpness of BD peaks has been significantly improved with the addition of optimized quadrupoles. With the quadrupole focusing system, the high beam charge increased the peak intensity and improved the resolution of the diffraction patterns significantly when compared with the original solenoid only system.

### Emittance measurement *via* BDI

The peak width of BDI is related to the divergence angle. Hence, it can be used to estimate the divergence angle, as explained in the following. The divergence angle can be obtained concurrently with the beam size measurement, taking only tens of seconds. This approach provides a way to measure emittance without a quadrupole scan. This is a significant advantage in the space charge dominated case. The quadrupole scan process may vary the emittance during the scan when the beam size changes due to the space charge effect. A typical quadrupole scan takes 10 minutes. Laser fluctuations and quadrupole power supply hysteresis are major error sources limiting the measurement precision.

The diffraction pattern formed by a single electron is described by Eq. (1),

1 $${I}_{n}={A}_{n}\cdot {A}_{n}^{\ast }$$

where *A*_*n*_ = *A*(*x*_*n*_, *y*_*n*_, *θ*_*n*_, *λ*_*n*_) is the wavefunction of the n^th^ electron, ${A}_{n}^{\ast }={A}^{\ast }({x}_{n},{y}_{n},{\theta }_{n},{\lambda }_{n})$ is the complex conjugate; *x*_*n*_ and *y*_*n*_ are the horizontal and vertical positions when the electron is incident on the sample, *θ*_*n*_ is the incident angle, n = 1, 2, …, *N*, *N* is the total number of electrons in the beam. The diffraction image formed by the entire electron beam can be approximated as,

2 $${I}_{tot}=\sum _{n=1}^{N}{I}_{n}$$

which is a superposition of the diffraction patterns formed by all the electrons. The number of electrons *N* is proportional to the beam charge. The distribution of the incident angle *θ* is determined by the beam emittance, and the spectral broadening can be calculated *via* the beam energy spread $\frac{{\rm{\Delta }}\lambda }{\lambda }=-\frac{{\rm{\Delta }}{\rm E}}{{\rm E}}$.

The width of the BD peak is determined by the peak broadening due to the sample properties, the energy spread of the electron beam, and its divergence. The peak broadening due to the sample properties is mainly determined by the grain size and strain of the poly-Au sample. In our case with the grain size of tens of nanometers and the DeBroglie wavelength of 3.8·10^−3^Å, it is about tens of µrad^16^. The energy spread of the electron beam causes the source spectral broadening, which is described by ${\rm{\Delta }}\theta =\,\tan (\theta )\cdot \frac{{\rm{\Delta }}\lambda }{\lambda }$. In the current setup, the (220) and (311) BD angle *θ* from the poly-Au sample are 1.33 mrad and 1.57 mrad. Their contributions to the BD broadening are several µrad (2–20 µrad). All other contributions are negligible compared to the contribution from the beam divergence, normally in the order of hundreds micro-radian. The divergence of the beam becomes the main factor determining the diffraction peak width. The beam divergence is obtained from the measured BD peak width. The electron beam size is directly measured at the sample position using a YAG screen with a resolution of 6.9 µm/pixel; therefore, the beam emittance can be determined with Eq. (3)^18^.

3 $${\varepsilon }_{nr}=\gamma \cdot {\sigma }_{r}\cdot {\sigma }_{r\text{'}}$$

**Figure 6:**
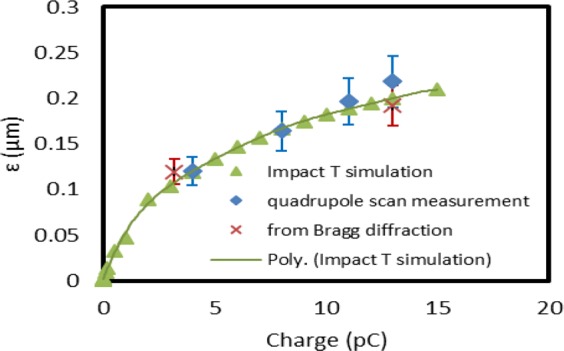
Beam emittance: measurement and simulation. The emittance measured by the BD method is represented by red crosses. The emittance simulated using the Impact-T is shown as green triangles. The emittance measured *via* standard quadrupole scan is plotted as blue diamonds.

Having a sufficient number of quadrupoles allows the beam waist with different beam sizes to be adjusted at the sample position. Therefore, the correlation term in the emittance equation^18^ becomes zero. The emittance measured by the BD method is represented by red crosses in Fig. 6. The emittance is simulated using the Impact-T code as a function of the beam charge. The gun phase was Ф_RF_ = 30° and laser spot size on the cathode was σ_x,y_ = 210 µm. The simulation results are plotted as green triangles. The emittances measured using standard quadrupole scans are plotted as blue diamonds. The errors are primarily coming from the shot-to-shot fluctuation of the laser energy, the uncertainty of the beam energy, the flag image resolution, and the beam aspect ratio. The beam energy has been independently measured by the magnet steering and BDI methods with an uncertainty of 8%.

When the charge increases from 1 to 13 pC, the emittance increases about 4 times. It indicates a fourfold increase of the divergence while the beam size is kept constant. Additionally, the energy spread of the beam increases from 1.5·10^−3^ to 1.3·10^−2^. In this condition, the resolution of ultrafast electron microscope increases linearly with the divergence of the beam^19^. The quadrupoles can keep either the size or the divergence of the beam nearly constant with varying the charge from 1 to 13 pC. We demonstrated that the beam divergence can be measured using the BDI method, as a bonus. For the future UEM experiment, the combination of quadrupole tunability and real-time measurement of the beam divergence allows the beam divergence to be kept constant at the optimal resolution while increasing the charge.

## Discussion

The high-charge, high-brightness, low-energy UED facility has been commissioned at ATF-II, BNL with the capability of generating 3.3 MeV electron bunches up to 13 pC charge (0.8·10^8^ electrons), 75 µm focused transverse beam size, and 1 ps bunch length. The charge density of the electron beam is about two orders of magnitude higher than what has been achieved previously using a solenoid only. Our proof-of-principal experiment has shown that when increasing the electron beam charge from 1 to 13 pC, the space-charge induced growth of the beam size can be compensated by online optimization of the quadrupoles.

The independent control of the beam size and divergence using quadrupole lenses has been successfully demonstrated and applied to improve the BDI intensity and resolution. A new method of measuring the beam divergence using BDI has been developed. We demonstrated the real-time measurement of the beam emittance. Our results agree well with simulations and with the traditional quadrupole scan.

Ultrafast electron microscopy, including diffraction, imaging, and spectroscopy, represents a unique opportunity for understanding structural dynamics and the behavior of matter under conditions far away from equilibrium at the required time and length scales. A successful quadruple-based transverse focusing system for higher charge density and smaller divergence of the electron beam, as the one we demonstrated here, can play a critical role in advancing the field and make UED and UEM more accessible to a broad scientific community. Compared to x-rays, the UED/UEM system will allow us to take advantage of the unique scattering power of electrons in the presence of space charge to discover emergent properties and dynamical behavior of exotic material systems and capture the response of biological molecules under applied stimuli.

## Methods

Our method to improve the UED resolution is based on a novel beam focusing system of quadrupole-multiplets. This transverse focusing system is broadly-tunable and highly efficient for online optimization of the electron beam to improve the BDI quality, which is determined by charge density and divergence of the beam at the sample. The divergence of the beam is usually larger than the peak broadening due to the sample properties. Therefore, the diffraction peak width can be used to measure the divergence and emittance of the beam. We call this the BDI method. The real-time divergence measurement opens the possibility of online optimization of the beam divergence (<0.2 mrad) at the sample with the increased beam charge.

## References

[1] Zhu P (2015). Femtosecond time-resolved MeV electron diffraction. *N. J*. Phys..

[2] He A, Willeke F, Yu LH (2014). Ultrashort x-ray pulse generation by electron beam slicing in storage rings. Phys. Rev. ST Accel. Beams..

[3] He A (2015). Design of low energy bunch compressors with space charge effects. Phys. Rev. ST Accel. Beams..

[4] Weathersby SP (2015). Mega-electron-volt ultrafast electron diffraction at SLAC National Accelerator Laboratory. Rev. Sci. Instrum..

[5] Fu F (2014). High quality single shot ultrafast MeV electron diffraction from a photocathode radio-frequency gun. Rev. Sci. Instrum..

[6] Musumeci P (2010). High quality single shot diffraction patterns using ultrafast megaelectron volt electron beams from a radio frequency photoinjector. Rev. Sci. Instrum..

[7] Li R (2009). Experimental demonstration of high quality MeV ultrafast electron diffraction. Rev. Sci. Instrum..

[8] Hastings JB (2006). Ultrafast time-resolved electron diffraction with megavolt electron beans. Appl. Phys. Lett..

[9] Chapman HN (2006). Femtosecond diffractive imaging with a soft-X-ray free-electron laser. Nat. Phys..

[10] Yang J (2016). Diffractive imaging of a rotational wavepacket in nitrogen molecules with femtosecond megaelectronvolt electron pulses. Nat. Commun..

[11] Lim J (2005). Adjustable, short focal length permanent-magnet quadrupole based electron beam final focus system. Phys. Rev. ST Accel. Beams..

[12] Xiang D (2014). Accelerator-based single-shot ultrafast transmission electron microscope with picosecond temporal resolution and nanometer spatial resolution. Nucl. Instrum. Methods Phys. Res. Sect. B..

[13] Huang X, Corbett J, Safranek J, Wu J (2013). An algorithm for online optimization of accelerators. Nucl. Instr. and Meth. A..

[14] Minty, M. Diagnostics. CERN Accelerator School. Internal report at, http://cds.cern.ch/record/798237/files/cm-p00050886.pdf (2004).

[15] Qiang J (2006). Three-dimensional quasistatic model for high brightness beam dynamics simulation. Phys. Rev. ST Accel. Beams..

[16] He K, Chen N, Wang C, Wei L, Chen J (2018). Method for Determining Crystal Grain Size by X-Ray Diffraction. Cryst. Res. Technol..

[17] Mo MZ (2016). Single-shot mega-electronvolt ultrafast electron diffraction for structure dynamic studies of warm dense matter. Rev. Sci. Instrum..

[18] Lejeune, C. & Aubert, J. Emittance and Brightness: Definitions and Measurements. Applied Charged Particle Optics, Part A. 159–259 (Academic Press, New York, 1980).

[19] Wan W, Chen F, Zhu Y (2018). Design of compact ultrafast microscopes for single- and multi-shot imagining with MeV electrons. Ultramicroscopy..

